# Oxford Nanopore Sequencing in pediatric emergency infectious diseases: from rapid diagnosis to precision medicine

**DOI:** 10.3389/fcimb.2026.1793808

**Published:** 2026-05-08

**Authors:** Yuzhe Guo, Anna Cheng, Yujuan Huang

**Affiliations:** Department of Emergency, Shanghai Children’s Hospital, School of Medicine, Shanghai Jiao Tong University, Shanghai, China

**Keywords:** host-pathogen interaction, Oxford Nanopore Sequencing, pediatric infectious diseases, point-of-care testing, precision medicine, rapid diagnostics

## Abstract

Pediatric emergency infectious diseases necessitate rapid and accurate etiologic diagnosis to inform timely, life-saving interventions. However, conventional diagnostic approaches are often hindered by prolonged turnaround times and limited capability in detecting diverse pathogens. Oxford Nanopore Technologies (ONT) sequencing, a fourth-generation, real-time genomic platform, offers a transformative solution. With unique features such as ultra-long read lengths, real-time data streaming, and portability, ONT enables comprehensive microbial characterization directly at the point of care, even from low-biomass clinical specimens. This review outlines the fundamental principles of ONT sequencing and recent advances in protocol optimization for challenging pediatric samples. We highlight its groundbreaking applications in acute respiratory, bloodstream, and central nervous system infections, demonstrating its capacity to simultaneously deliver pathogen identification, antimicrobial resistance profiling, and strain typing. Moreover, ONT facilitates novel insights into host-pathogen interactions through integrated genomic and transcriptomic analyzes. We also address current challenges, including bioinformatics complexity and analytical standardization, and propose pathways toward integration within a precision infectious disease framework. Beyond diagnostics, ONT is emerging as a powerful tool to advance infection biology and improve clinical outcomes in children.

## 1 Introduction

Infectious diseases remain a leading cause of morbidity and mortality among children worldwide (Khan et al., 2022), particularly in emergency and critical care settings. In pediatric patients, clinical manifestations are often non-specific, yet disease progression can be alarmingly rapid (Zhang et al., 2023). Life-threatening conditions such as sepsis, meningitis, and severe pneumonia necessitate prompt initiation of targeted antimicrobial therapy. Accumulating evidence indicates that in sepsis and meningitis, every hour of delay in effective antimicrobial administration is associated with significantly increased mortality (Lane et al., 2024; Martín-Cerezuela et al., 2023). This clinical urgency underscores an unmet need for diagnostic strategies capable of delivering timely and accurate etiologic identification-ideally within hours, rather than days.

Despite advances in laboratory medicine, current diagnostic approaches fall short of this benchmark. Culture-based methods, while historically regarded as the gold standard for many bacterial infections, are time-consuming, typically requiring 24–72 hours for results (Tang et al., 2021), and demonstrate limited sensitivity for fastidious organisms, viruses, and fungi (Gu et al., 2025; Cirilli et al., 2024; Vanbiervliet et al., 2025). Molecular assays such as multiplex PCR panels have shortened turnaround times but are constrained by their reliance on pre-defined, narrow pathogen menus (Robert-Gangneux et al., 2025; Caldwell et al., 2025). These platforms are poorly suited for detecting novel, unexpected, or co-infecting pathogens-scenarios frequently encountered in critically ill children, particularly among immunocompromised individuals or those with underlying comorbidities.

Metagenomic next-generation sequencing (mNGS) represented a major leap forward by enabling hypothesis-free, broad-spectrum pathogen detection directly from clinical specimens. However, its dependence on short-read sequencing technologies poses notable limitations for infectious disease diagnostics (Buddle et al., 2024; Lazarevic et al., 2022). Short reads often fail to reconstruct complete microbial genomes, especially in the presence of repetitive sequences (Sim et al., 2022) or high GC content (Orellana et al., 2023). More importantly, they frequently lack the resolution to localize antimicrobial resistance (AMR) genes and virulence factors within their genomic context (Rodino and Simner, 2024; Govender et al., 2021; Serpa et al., 2022). Since these elements are commonly carried on mobile genetic elements such as plasmids, distinguishing between chromosomal and transmissible resistance mechanisms is essential for understanding transmission dynamics and outbreak risks-an analytical nuance difficult to achieve with short-read data (Wang et al., 2022; Zongo et al., 2024).

This substantial gap-between the imperative for rapid, comprehensive diagnosis and the constraints of current sequencing paradigms—has spurred interest in next-generation platforms with enhanced capabilities. Oxford Nanopore Technologies (ONT) sequencing has emerged as a uniquely promising solution. As a fourth-generation, single-molecule, real-time approach, ONT enables direct, real-time analysis of native DNA/RNA from clinical specimens, offering ultra-long read lengths, portable deployment, and minimal infrastructure requirements (Dyshlovoy et al., 2024; Mostafa, 2024). These attributes position ONT as a viable near-patient or point-of-care tool, with the potential to dramatically compress diagnostic timelines from days to hours, thereby transforming the management of pediatric emergency infections.

In this review, we provide a comprehensive overview of the burgeoning role of ONT in this diagnostic revolution. We outline the technological foundations of ONT, assess its clinical performance across major pediatric infection syndromes, highlight its emerging utility in dissecting host-pathogen interactions, and discuss current challenges and strategic directions toward routine implementation in clinical practice.

## 2 Technological principles of Oxford Nanopore Sequencing and optimizations for clinical diagnostics

The transformative potential of ONT in clinical microbiology arises from its fundamental departure from conventional sequencing paradigms. Unlike technologies that rely on PCR amplification or fluorescent nucleotide labeling, ONT enables the direct, real-time analysis of native DNA and RNA molecules at the single-molecule level (Xie et al., 2021). This unique capability positions ONT as a disruptive tool for rapid pathogen detection and genomic characterization in clinical settings, particularly in pediatric emergencies where diagnostic speed is paramount.

### 2.1 Core sequencing principle

**Figure 1:**
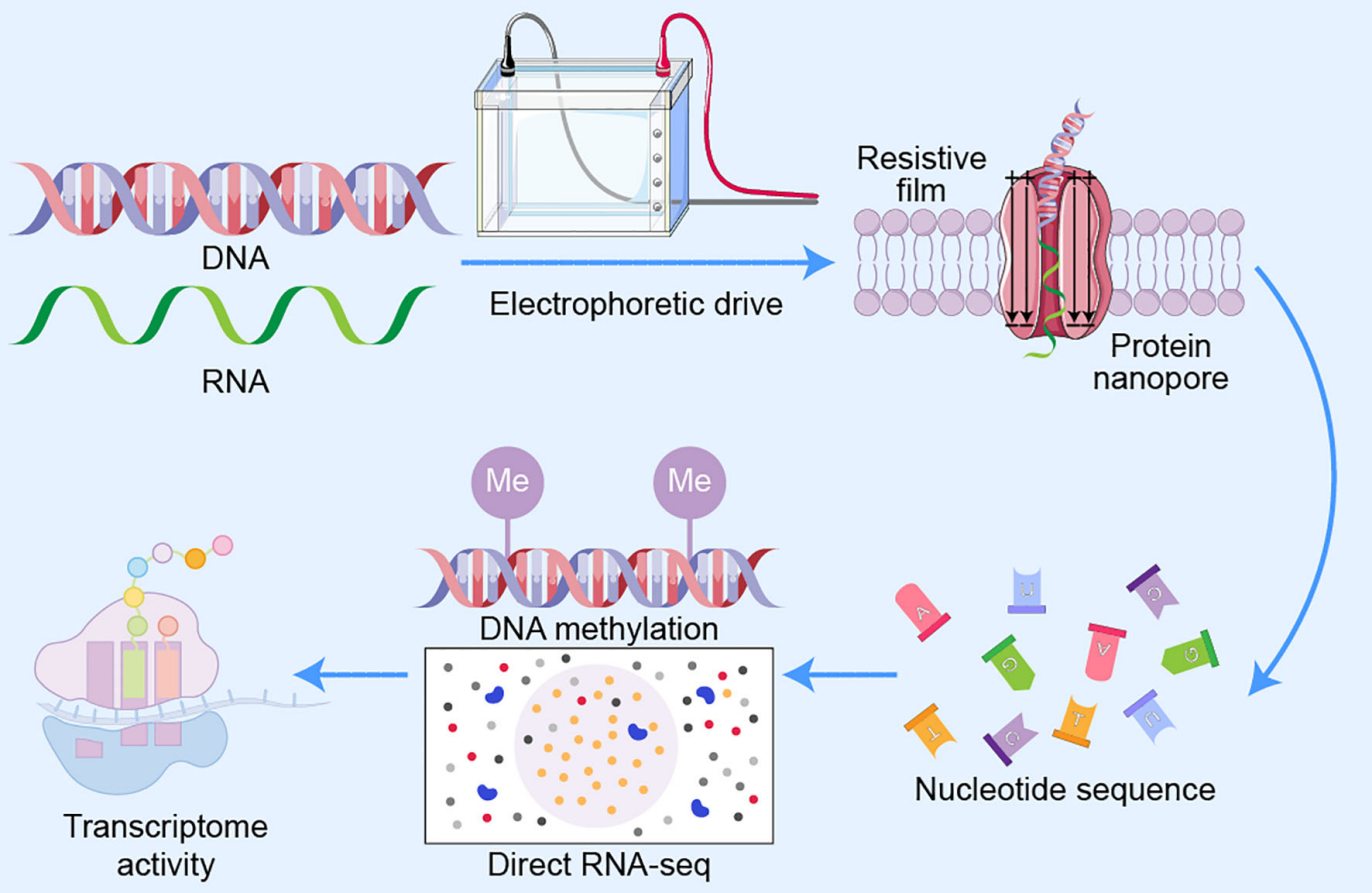
Schematic illustration of the Oxford Nanopore Technologies (ONT) sequencing principle. Schematic representation of the core mechanism of ONT sequencing. Single-stranded DNA or RNA molecules are electrophoretically driven through a protein nanopore embedded in an electrically resistant membrane. As each nucleotide passes through the pore’s narrowest constriction (the α-hemolysin or CsgG-like channel), it induces a characteristic disruption in the ionic current, which is recorded in real time. Sophisticated base-calling algorithms translate these electrical signals into nucleotide sequences. This method enables direct detection of native nucleic acid modifications (e.g., DNA methylation) and supports direct RNA sequencing (direct RNA-seq), offering simultaneous insights into pathogen and host transcriptomic activity during infection.

ONT is based on the principle of electrophoretically driven translocation of nucleic acids through biologically engineered nanopores-protein channels embedded within an electro-resistant synthetic membrane. Application of a constant voltage bias across the membrane drives single-stranded DNA or RNA through a nanopore. As each nucleotide traverses the channel’s narrowest region, it induces a transient, base-specific disruption in the ionic current, generating a unique electrical signature (Tounsi et al., 2022; Genner et al., 2025). These signals are captured in real time and interpreted by machine learning–based base-calling algorithms to infer the nucleotide sequence (**Figure 1**).

A key advantage of this approach lies in its ability to preserve native nucleic acid modifications during sequencing. For example, DNA methylation—a critical regulator of bacterial virulence and epigenetic regulation—can be directly detected without additional processing. Moreover, ONT supports direct RNA sequencing, offering an unprecedented opportunity to profile pathogen transcriptomes and host immune responses concurrently during infection (Genner et al., 2025; Rahman et al., 2021).

### 2.2 The MinION platform and evolution of performance

The MinION is Oxford Nanopore Technologies’ (ONT) flagship device, roughly USB−stick sized and integrating all sequencing components. Powered and connected via a standard USB interface, it enables operation outside centralized labs—in resource−limited field sites and at patient bedsides—collapsing conventional diagnostic timelines (Tounsi et al., 2022; Peel et al., 2025). A single flow cell can generate tens of gigabases per run (Katsman et al., 2022). Advances in pore chemistry and kits have markedly improved accuracy. The R10.4.1 pore with Kit ;14 chemistry delivers simplex read accuracies >Q20 (>99%) and duplex consensus accuracies approaching Q30 (>99.9%), meeting clinical standards for variant calling and antimicrobial resistance (AMR) profiling (Mumm et al., 2023; Ye et al., 2024).

Nanopore sequencing relies on single−molecule real−time (SMRT) principles: as nucleic acids pass through nanoscale pores under an electric field, ionic current fluctuations reveal nucleotide identity and chemical modifications. This eliminates the need for PCR amplification or bisulfite conversion, preserving native epigenetic information (Dyshlovoy et al., 2024). Modified bases alter conformational dynamics and charge distribution, producing distinct current signatures distinguishable from unmodified sequences (Bertocchi et al., 2025). ONT platforms detect diverse marks—including 5−mC, 5−hmC, and 6−mA—at single−molecule resolution, enabling concurrent acquisition of sequence and modification data (Wang et al., 2021).This multimodal, single−molecule capability allows simultaneous profiling of genomic variation, transcript structure, and epigenetic landscapes, providing a robust foundation for multidimensional molecular analysis of complex diseases, as demonstrated in brain tumor research (Dyshlovoy et al., 2024).

### 2.3 Key advantages for infectious disease diagnostics

**Figure 2:**
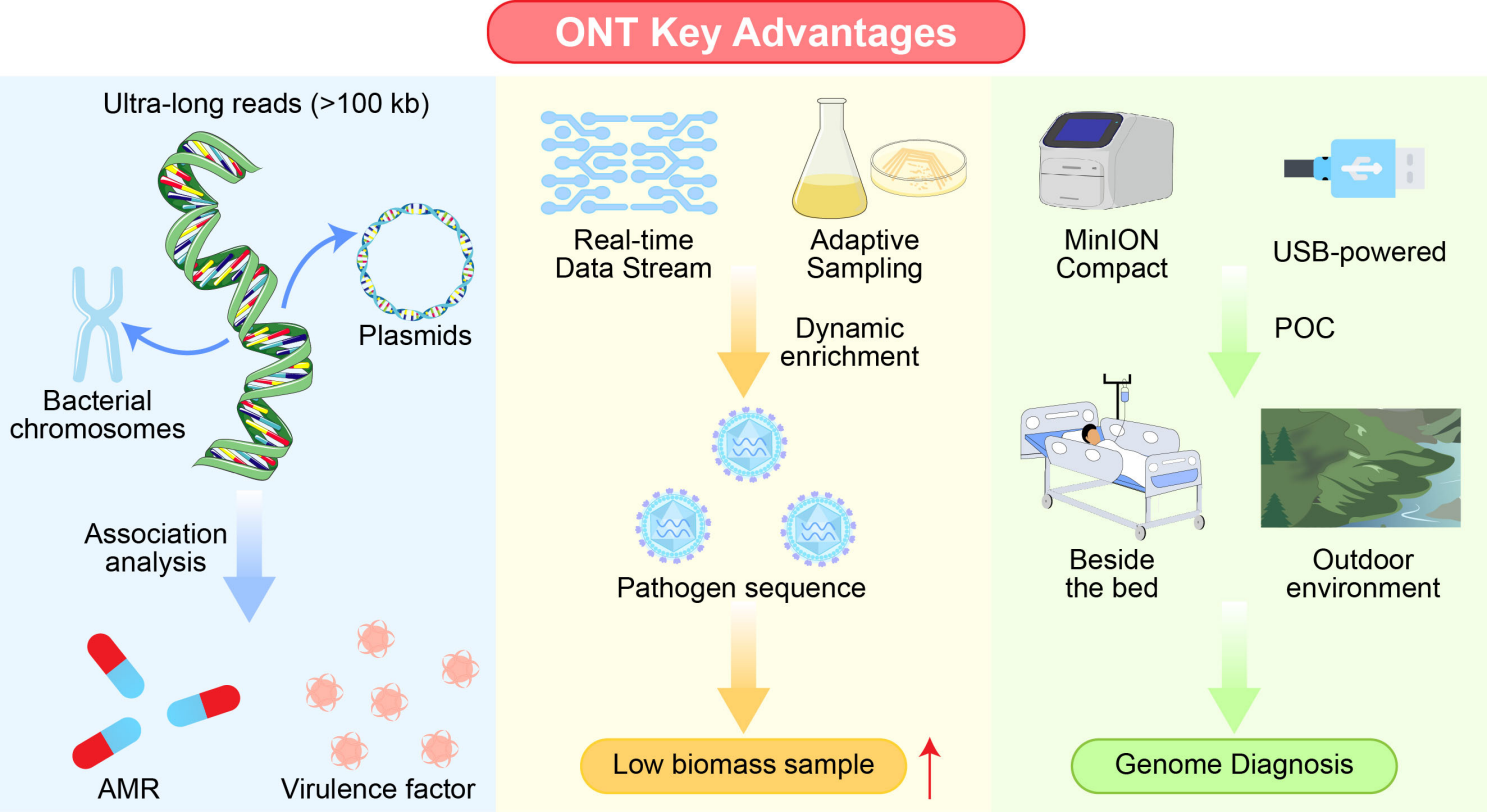
Key technological advantages of ONT for infectious disease diagnostics. Illustrated overview of three defining strengths of ONT sequencing in clinical microbiology. Ultra-long read lengths (>100 kb) enable *de novo* assembly of complete bacterial chromosomes and plasmids, facilitating linkage analysis of antimicrobial resistance (AMR) genes and virulence factors. Real-time data streaming and adaptive sampling allow dynamic enrichment of pathogen sequences and early detection during ongoing runs, enhancing performance in low-biomass samples. MinION’s compact, USB-powered design supports true point-of-care (POC) use, enabling genomic diagnostics outside traditional laboratory environments, including bedside or field settings.

Three distinctive features make ONT especially well-suited for managing infectious diseases in pediatric emergency settings (**Figure 2**):

#### 2.3.1 Ultra-long read lengths

ONT routinely generates reads exceeding 100 kilobases (kb), enabling contiguous, gapless assembly of complex genomes. This is particularly advantageous for resolving repetitive genomic regions and assembling complete bacterial chromosomes and, crucially, extrachromosomal elements such as plasmids (Zidane et al., 2025; Mumm et al., 2023). Long reads facilitate the physical linkage of AMR genes, virulence factors, and mobile genetic elements within a single sequencing read, allowing comprehensive characterization of a pathogen’s functional repertoire in a single assay (Biggel et al., 2024).

#### 2.3.2 Real-time data streaming and adaptive sampling

ONT acquires and analyzes data continuously during a sequencing run. This permits preliminary pathogen identification and AMR detection within minutes to hours of initiating sequencing (Smith et al., 2020; Campodónico et al., 2025). Additionally, real-time analysis enables “adaptive sampling,” a bioinformatic strategy that enhances pathogen signal by selectively retaining or rejecting molecules as they pass through the pore. Host-derived or off-target reads are identified on-the-fly and ejected by reversing the voltage, thereby enriching the output for microbial sequences (De Meulenaere et al., 2024; Lin et al., 2023). This feature is especially valuable for low-biomass samples such as cerebrospinal fluid (CSF) or blood, where human DNA overwhelmingly predominates.

#### 2.3.3 Portability and streamlined workflow

MinION’s portability democratizes access to sequencing, making genuine point-of-care (POC) or near-patient diagnostics feasible (Wallace et al., 2023; Sheka et al., 2021). When coupled with rapid, field-compatible nucleic acid extraction and library preparation kits, the entire workflow—from clinical specimen to interpretable genomic report—can be completed within a single clinical shift in emergency departments (Xiao et al., 2025), directly addressing the critical need for expeditious decision-making.

### 2.4 Optimized protocols for low-biomass and complex samples

Pediatric clinical specimens, such as CSF, whole blood, or bronchoalveolar lavage fluid, are often characterized by small volumes and low microbial burden, posing challenges for sensitive detection. To overcome this, specialized protocols have been developed to minimize host background and enhance microbial signal. For instance, targeted metatranscriptomic sequencing approaches (e.g., mtTGS) exploit the abundance of microbial ribosomal RNA (rRNA) in metabolically active cells to preferentially capture pathogen-derived transcripts (Fu et al., 2023). Such targeted strategies markedly improve sensitivity in difficult matrices and are pivotal for translating ONT’s technological promise into robust, reproducible clinical workflows.

## 3 Clinical applications in pediatric emergency infectious diseases

**Figure 3:**
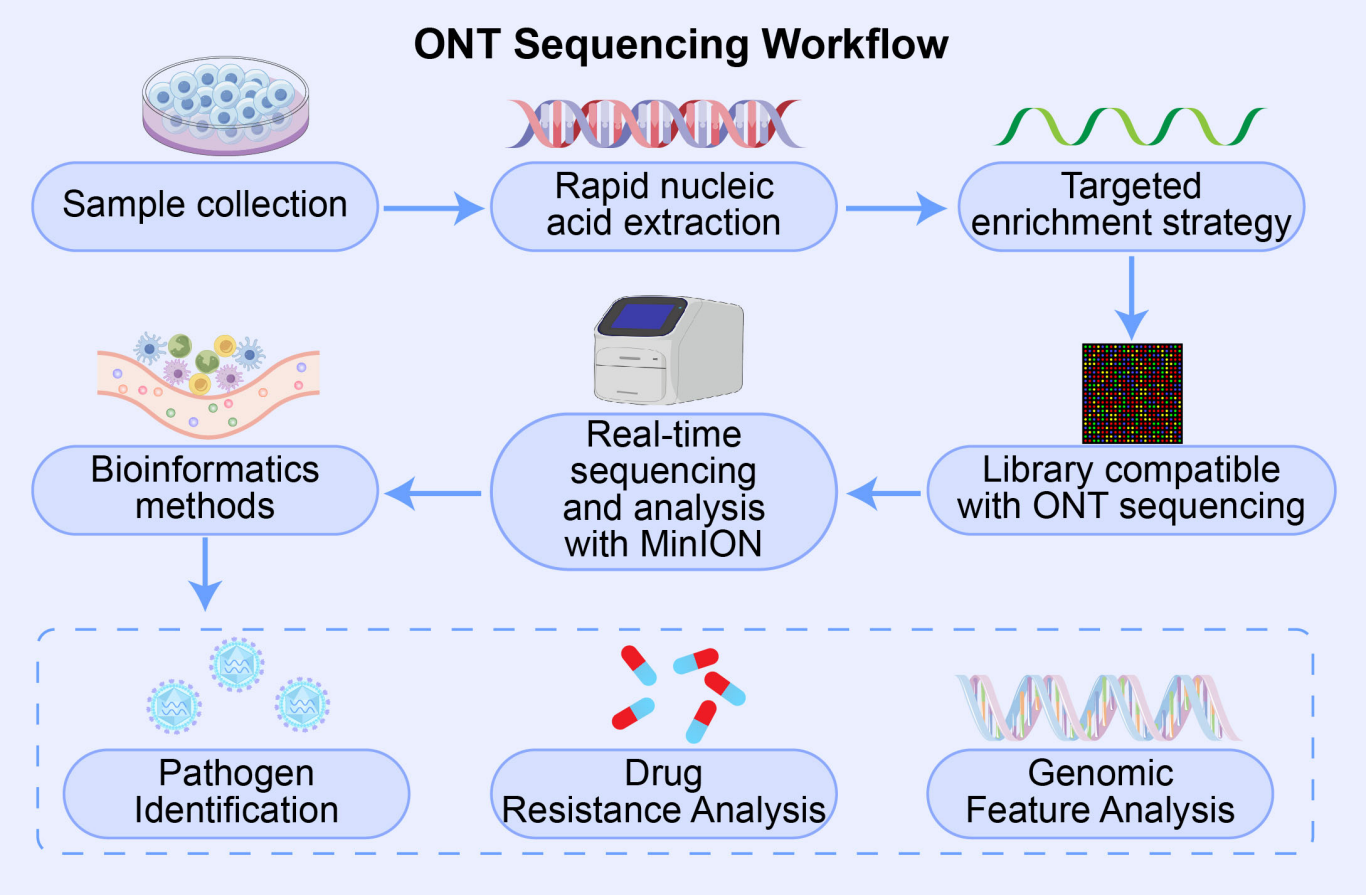
Optimized ONT workflow for low-biomass pediatric clinical specimens. Proposed workflow for applying ONT sequencing to pediatric samples with limited volume and microbial load (e.g., cerebrospinal fluid, blood, bronchoalveolar lavage). Steps include: (1) sample collection, (2) rapid nucleic acid extraction, (3) implementation of targeted enrichment strategies (e.g., rRNA-based mtTGS) to suppress host background, (4) library preparation compatible with ONT sequencing, (5) real-time sequencing and analysis using MinION, and (6) bioinformatics interpretation for pathogen identification, AMR profiling, and genomic characterization. This streamlined pipeline facilitates translation of ONT technology into routine pediatric emergency diagnostics.

The unique capabilities of Oxford Nanopore Technologies (ONT) are increasingly being leveraged to address longstanding diagnostic challenges across a spectrum of severe pediatric infectious diseases. From respiratory and bloodstream infections to central nervous system (CNS) pathologies, ONT is enabling rapid, comprehensive, and actionable genomic insights that are transforming clinical decision-making at the point of care (**Figure 3**).

### 3.1 Acute respiratory infections

Community-acquired and ventilator-associated pneumonia in children pose considerable diagnostic complexity due to the involvement of diverse pathogens, including bacteria, viruses, and atypical agents. ONT-based metagenomic sequencing (ONT-mNGS) enables culture-independent, hypothesis-free pathogen detection directly from nasopharyngeal swabs or bronchoalveolar lavage fluid. Studies have demonstrated that ONT-mNGS can identify not only canonical respiratory pathogens such as Streptococcus pneumoniae, Mycoplasma pneumoniae, and respiratory viruses, but also uncover mixed infections and rare or unexpected organisms overlooked by conventional multiplex PCR panels (Khan et al., 2022).

A key strength of ONT lies in its ability to generate ultra-long reads, facilitating high-resolution strain typing and molecular epidemiology investigations. For instance, ONT has enabled discrimination between community-acquired and healthcare-associated methicillin-resistant S. aureus (MRSA) strains during outbreak investigations (Landman et al., 2024; Bogaerts et al., 2024). Furthermore, long-read sequencing allows direct detection of antimicrobial resistance (AMR) determinants—such as mecA in S. aureus and β-lactamase genes in Gram-negative bacilli—thereby guiding timely escalation or de-escalation of empirical antibiotic therapy.

### 3.2 Bloodstream infections and sepsis

Sepsis represents one of the most time-sensitive conditions in pediatric emergency medicine, where delays in appropriate antimicrobial therapy are strongly associated with adverse outcomes (Lane et al., 2024). Although blood culture remains the clinical standard, its utility is constrained by slow turnaround times and frequent false negatives, particularly in neonates and immunocompromised hosts.

ONT sequencing of cell-free DNA (cfDNA) from plasma or direct analysis of positive blood culture broths offers a promising near-patient alternative. Pioneering studies, including investigations into gut microbiota development and sepsis susceptibility in preterm infants, have illustrated that ONT can detect causative pathogens and delineate their AMR profiles within hours of sample processing (Liu et al., 2024). In neonatal intensive care units (NICUs), for example, ONT has facilitated the rapid identification of carbapenem-resistant Klebsiella pneumoniae outbreak strains and associated resistance genes such as blaKPC, enabling swift infection control interventions and precision-guided therapy (Koch et al., 2023). Importantly, ONT’s real-time data streaming allows sequencing to be terminated upon reaching diagnostic confidence, minimizing unnecessary run time and expediting actionable reporting.

### 3.3 Central nervous system infections

Meningitis and encephalitis are neurological emergencies in children, carrying substantial risks of mortality and long-term neurologic sequelae. Cerebrospinal fluid (CSF) specimens are typically characterized by minute microbial loads, complicating pathogen detection by conventional methods.

ONT-based sequencing—particularly when integrated with targeted enrichment strategies or adaptive sampling—has shown encouraging success in this context (Alvarez Mulett et al., 2025; Kinsella et al., 2022). It enables sensitive detection of diverse neurotropic pathogens, including enteroviruses, herpes simplex virus, Streptococcus agalactiae, Neisseria meningitidis, and Cryptococcus neoformans, from small-volume CSF samples. The concurrent profiling of AMR genes is especially valuable in bacterial meningitis, where prompt recognition of resistance mechanisms (e.g., penicillin resistance in S. pneumoniae) can prompt immediate therapeutic adjustments.

Moreover, direct RNA sequencing of CSF opens up the possibility of profiling host neuroinflammatory responses, potentially yielding prognostic biomarkers linked to disease severity and outcome (Katopodi et al., 2025). Thus, ONT expands the diagnostic scope beyond pathogen identification to encompass host-pathogen interaction dynamics in CNS infections.

### 3.4 Beyond pathogen identification: delivering actionable genomic intelligence

The clinical utility of ONT transcends binary “pathogen-detected-or-not” outputs. Three principal features enable it to deliver high-value, clinically actionable information:

#### 3.4.1 Comprehensive AMR profiling

By enabling gapless assembly of plasmids and other mobile genetic elements, ONT allows precise mapping of AMR genes within their genomic and epidemiological contexts. This distinction between chromosomally encoded and horizontally transferred resistance mechanisms is critical for assessing transmission potential and designing containment strategies (Lipworth et al., 2024; Ye et al., 2024).

#### 3.4.2 Virulence typing and strain discrimination

ONT facilitates the identification of virulence factors (e.g., streptococcal pyrogenic exotoxins, staphylococcal enterotoxins) and supports high-resolution strain typing via core genome multilocus sequence typing (cgMLST) or single-nucleotide polymorphism (SNP) analysis. These capabilities markedly enhance outbreak tracing and phylogenetic resolution compared to conventional typing methods (Baktash et al., 2022; Guérin et al., 2025).

#### 3.4.3 Dissection of polymicrobial infections

Many pediatric infections, such as those seen in cystic fibrosis–related pneumonia or complicated intra-abdominal infections, involve complex polymicrobial communities. ONT’s long reads permit independent assembly of constituent genomes, unraveling the structure and composition of the infectious consortium with high fidelity (Agustinho et al., 2024; Orellana et al., 2023). This systems-level view informs both therapeutic targeting and ecological understanding of microbial interactions.

Beyond its diagnostic utility, ONT also provides a powerful platform for investigating the dynamic interplay between host and pathogen, a frontier we explore in the following section.

### 3.5 Clinical performance validation and application advantages of ONT sequencing in pediatric infectious diseases

#### 3.5.1 ONT sequencing in pediatric respiratory tract infections: clinical efficacy validation and application advantages

In recent years, an increasing number of clinical investigations-particularly prospective studies utilizing pediatric respiratory tract infection specimens-have systematically evaluated the diagnostic performance of nanopore sequencing in real-world settings, highlighting its tangible value in guiding precision antimicrobial therapy. Collectively, these studies demonstrate that ONT platforms not only achieve high−sensitivity detection across a broad pathogen spectrum, but also furnish clinically actionable information that directly informs therapeutic decision−making. Specifically, ONT sequencing enables simultaneous identification of antimicrobial resistance determinants, delineation of polymicrobial infections, and reconstruction of nosocomial transmission pathways, thereby facilitating rapid, evidence−based interventions and enhancing infection control strategies in pediatric care.

##### 3.5.1.1 Mixed infections and drug resistance gene detection

A prospective multicenter study by Guo et al (Guo et al. 2023). employed a Meta−Panel analytical workflow with ONT sequencing to investigate 450 pediatric respiratory specimens. This approach enabled simultaneous detection of bacteria, fungi (with the exception of Aspergillus spp.), and viruses, achieving an overall sensitivity of 80.2% and a specificity of 98.8%. Beyond pathogen identification, the methodology accurately resolved the etiologic source of each microorganism in polymicrobial infections and concurrently detected clinically relevant resistance determinants-such as bla and mecA-within mixed infection contexts. This capacity for integrated pathogen–resistance profiling facilitates precise microbial typing and informs tailored therapeutic regimens for complex pediatric respiratory infections, thereby bridging molecular diagnostics with precision antimicrobial stewardship.

##### 3.5.1.2 Typing, drug resistance, and epidemiological tracking

The capacity of ONT sequencing for pathogen typing, provenance tracing, and nosocomial infection surveillance has been substantiated in clinical settings. In a United Kingdom study during the 2018–2019 influenza season, Xu et al (Xu et al. 2021). performed ONT metagenomic sequencing on 180 respiratory specimens. For influenza A virus detection, ONT achieved a sensitivity of 83% (75/90) and a specificity of 93% (84/90). Beyond pathogen identification, the platform simultaneously detected the oseltamivir−resistant substitution S331R and delineated nosocomial transmission clusters, alongside identification of other co−circulating pathogens. These findings highlight ONT’s unique potential as an integrated “one−stop” solution for concurrent diagnosis, resistance profiling, and epidemiological tracking-thereby offering a powerful tool for real−time outbreak containment and precision infection control in pediatric care settings.

##### 3.5.1.3 Rapid turnaround to guide antibiotic adjustment

The clinical translational value of ONT sequencing in expediting diagnosis and informing antimicrobial therapy has been demonstrated in a prospective pilot study conducted in a pediatric intensive care unit (PICU). Baldan et al (Baldan et al. 2021). applied nanopore 16S rRNA gene sequencing (Np16S) to respiratory specimens from PICU patients, achieving correct strain identification in 84% of cases within 1 ;hour and detecting pathogens in 97% (32/33) of culture−positive samples. Concordance with conventional culture results reached 80.5%.

The most immediate clinical impact of this rapid turnaround lies in antimicrobial stewardship: up to 67% (30/45) of patients received timely, targeted antibiotic regimen adjustments based on ONT−derived results. These findings provide compelling evidence that ONT, as a near−patient or rapid−response molecular platform, can markedly reduce the interval from sample collection to effective treatment initiation, thereby enhancing outcomes in critically ill children and supporting precision infection management in high−acuity settings.

#### 3.5.2 Key performance and application value of ONT sequencing based on clinical data in pediatric bloodstream infections

Multiple large−scale, prospective clinical evaluations have demonstrated that ONT–based assays provide substantial gains in pathogen detection sensitivity for bloodstream infections compared with the gold−standard blood culture.

##### 3.5.2.1 Improved pathogen detection and diagnostic efficiency

ONT–based targeted sequencing demonstrates clear technical superiority over conventional blood culture in both pathogen identification and diagnostic throughput. In a prospective study of 387 blood specimens, Han et al (Han et al. 2024). utilized NTS and observed a pathogen positivity rate of 69.5%, substantially higher than the 33.9% achieved by traditional blood culture. NTS achieved a sensitivity of 84.0% and a specificity of 90.1%, with an intermethod agreement of 90.2% when compared to mNGS. Notably, the entire NTS workflow was completed in approximately 7 ;hours, highlighting its capacity to expedite pathogen identification in pediatric bacteremia and sepsis. These findings were corroborated by a study of 50 children with hematological malignancies and severe infections (Zhang et al., 2023), which reported an NTS pathogen detection rate of 90%-far surpassing rates obtained by blood culture (32.6%) and anal swab screening (14.6%). Collectively, these data establish NTS as a rapid, high−yield alternative to conventional diagnostics, enabling detection of clinically relevant organisms that may be overlooked or delayed by standard culture−based approaches.

##### 3.5.2.2 Guiding precise antibiotic adjustment to improve outcomes

The clinical translational value of ONT sequencing following pathogen and antimicrobial resistance identification has been substantiated by accumulating data, with its core impact lying in improving patient outcomes through timely treatment optimization. In the aforementioned study of children with hematological malignancies (Zhang et al., 2023), clinicians adjusted antibiotic regimens promptly based on definitive etiologic evidence provided by NTS. Among 43 children who underwent regimen modification guided by NTS results, the treatment response rate reached 93.02% (40/43), and notably, no infection−related deaths occurred. These findings strongly indicate that NTS can effectively inform clinical decision−making and markedly reduce infection−associated mortality in high−risk pediatric populations.

Further supporting this paradigm, a central Taiwan study (Liu et al., 2024) applied ONT sequencing combined with an adaptive sampling strategy to 458 positive blood culture samples. The approach enabled accurate species identification of 76 common pathogens-including Escherichia coli, Klebsiella pneumoniae, Staphylococcus aureus, and Candida spp.-while also achieving comprehensive resistance profiling. Using a modified ResFinder database, predictions of antimicrobial resistance in monobacterial infections demonstrated >90% concordance with reference methods. This rapid, integrated “pathogen–resistance” output provides clinicians with a direct basis for early and precise antibiotic adjustment, thereby facilitating individualized therapy and improved clinical outcomes.

#### 3.5.3 Key performance and application value of ONT sequencing in pediatric central nervous system infections

Central nervous system (CNS) infections represent a life−threatening condition in pediatric practice. Prompt and accurate etiologic diagnosis is crucial for improving patient outcomes. Conventional diagnostic approaches are often hampered by limited sensitivity and prolonged turnaround times. Targeted nanopore sequencing (tNPS) has emerged as a valuable clinical tool, leveraging its distinctive technical attributes to overcome these limitations.

##### 3.5.3.1 High sensitivity and specificity detection

tNPS employs specific primer−based enrichment or hybrid capture to concentrate pathogen sequences, substantially enhancing detection sensitivity while preserving high analytical throughput-an advantage particularly relevant to pediatric CNS infection diagnostics. In a multicenter prospective study conducted in China (Chen et al., 2024), 152 children with suspected CNS infections were enrolled. Pathogen−targeted nanopore sequencing (ptNGS) achieved a pathogen detection rate of 65.1%, significantly higher than that of mNGS (47.4%) and conventional methods. Positive percent agreement (PPA) and negative percent agreement (NPA) for ptNGS were 64% and 66.7%, respectively, outperforming mNGS and underscoring the robust performance of tNPS in pathogen identification.

##### 3.5.3.2 Rapid diagnosis wins intervention time window

Beyond its high sensitivity, tNPS offers rapid turnaround, securing a critical intervention window during the acute phase of disease. The entire workflow-from sample receipt to reporting-can be completed within 6-7 ;hours, substantially faster than conventional culture or standard mNGS approaches (Han et al., 2024; Zhang et al., 2023). For children with CNS infections characterized by rapid clinical deterioration, timely etiologic diagnosis enables prompt initiation of targeted therapies and may markedly reduce the risk of long−term neurological sequelae (**Table 1**).

**Table 1:** Clinical performance validation and application advantages of Oxford Nanopore Sequencing in pediatric infectious diseases.

Fields of application	Related research	Clinical value
Acute respiratory infections	Prospective study with 450 respiratory samples from children (Meta-Panel joint analysis method) (Guo et al., 2023)	1.High sensitivity and broad spectrum detection: 80.2% sensitivity and 98.8% specificity for bacteria, viruses, fungi and other pathogens. 2. Simultaneous identification of mixed infections and drug resistance genes : Co-infections can be detected, and key drug resistance genes such as bla and mecA can be found simultaneously
	UK 180 samples influenza season study (2018-2019) (Xu et al., 2021)	Typing and nosocomial Infection Surveillance: Achieve accurate influenza H1/H3 typing and identify clusters of nosocomial transmission
	A prospective pilot study of respiratory tract samples in PICU (Baldan et al., 2021)	Rapid guided treatment: About 67% of PICU children adjusted antibiotics based on ONT results, significantly improving treatment targeting
Bloodstream infections and sepsis	387 samples Prospective NTS Study (Han et al., 2024)	Significantly improved diagnostic rate: the detection rate of NTS was 69.5%, and the specificity was 90.1%, which was higher than the 33.9% positive rate of traditional blood culture
	Severe infection in 50 children with hematological diseases (Zhang et al., 2023)	Improved clinical outcomes: Treatment response rates reached 93.02% in high-risk children, with no infection-related deaths in the study
	A study of 458 positive blood cultures using ONT technology combined with adaptive sampling strategy in Taiwan (Liu et al., 2024)	1. Comprehensive identification of pathogens: effective detection of bacteria, fungi, viruses 2. High accuracy drug resistance prediction: Positive blood culture samples have a drug resistance prediction consistency >90%, supporting early precision medicine
Central nervous system infections	Multicenter prospective study of 152 Chinese patients (Chen et al., 2024)	High detection rate of low-biomass samples: ptNGS 65.1%, better than the traditional method (47.4%)
	Single-center prospective study of 68 cases of CNS infections Hybrid capture enhanced sequencing (Piantadosi et al., 2021)	Improved detection of difficult pathogens: Successful detection of Borrelia burgdorferi, Anaplasma, Powassan virus, and others by targeted enrichment

## 4 ONT in elucidating host-pathogen interactions: a new frontier

Conventional approaches to studying host–pathogen interactions have been constrained by limited technical resolution, low detection throughput, and restricted analytical dimensionality, often restricting investigation to a single biological compartment (host or pathogen) (Yang et al., 2021) and to static snapshots of molecular events (Yu et al., 2022). Consequently, these methods fail to capture the dynamic molecular alterations that occur throughout the course of infection, impeding a comprehensive understanding of the complex mechanisms underlying host–pathogen interplay (Yang et al., 2021; Yu et al., 2022).

A defining strength of ONT sequencing lies in its unique ability to resolve the dynamic, bidirectional crosstalk between host and pathogen at unprecedented resolution, positioning it at the forefront of contemporary cellular and infection microbiology research (Chang et al., 2022). By transcending static genomic profiling, ONT enables real−time molecular surveillance of infection processes, yielding transformative insights into disease pathogenesis and opening new avenues for mechanistic exploration.

### 4.1 Profiling the host transcriptional response in real time

Conventional methods for profiling the host transcriptional response following infection—such as reverse transcription PCR (RT-PCR) and short-read RNA sequencing—require reverse transcription and PCR amplification, steps that can introduce sequence bias and preclude full-length mRNA capture. These limitations hinder fine-grained, real-time analysis of immune response gene expression patterns (Gleeson et al., 2022; Gallardo et al., 2022). In addition, short-read platforms struggle to resolve complex transcript architectures, including alternative splicing events, thereby constraining in-depth mechanistic interrogation of host immunity (Hu et al., 2021; Su et al., 2023).

Direct RNA sequencing with ONT circumvents the inherent biases of reverse transcription and amplification, enabling native capture of full-length host messenger RNAs (Sun et al., 2025). When applied to clinical samples from infected children—such as whole blood or tissue biopsies—this approach allows granular, real-time profiling of the host immune response. By detecting specific cytokine or interferon-stimulated gene expression signatures, ONT can aid in distinguishing bacterial from viral etiologies, predicting disease severity, and dynamically monitoring therapeutic efficacy (Rao et al., 2022). For example, an ONT-derived “sepsis transcriptome” signature may furnish critical prognostic insights that extend well beyond pathogen identification, thereby informing precision medicine strategies.

### 4.2 Dissecting pathogen adaptation and evolution within the host niche

Conventional approaches to studying pathogen evolution in the host-such as short−read sequencing and genotyping-are constrained by read length limitations, precluding gapless genome assembly and impeding accurate detection of low−frequency adaptive mutations. These methods also fail to track dynamic changes in mobile genetic elements (e.g., plasmids and transposons) (Arredondo-Alonso et al., 2021) and lack the resolution needed to dissect microevolutionary processes underlying pathogen resistance and virulence modulation (Ortiz et al., 2022; Tonkin-Hill et al., 2025). Moreover, most traditional strategies rely on endpoint measurements, preventing real−time, longitudinal monitoring of evolutionary trajectories.

The long−read capability of ONT is uniquely suited to resolving microbial evolution as it occurs. In chronic infections-such as those seen in children with cystic fibrosis-serial sampling combined with ONT sequencing enables detailed tracking of pathogen microevolution within the lung microenvironment. For example, Pseudomonas aeruginosa populations can be monitored over time to reveal emergent adaptive mutations that confer antibiotic resistance, give rise to hypermutator phenotypes, or reshape virulence gene expression in direct response to host immune pressures and antimicrobial treatments (Cramer et al., 2023; Long et al., 2021). Thus, ONT provides an indispensable platform for elucidating mechanisms of microbial persistence and the molecular basis of treatment failure.

### 4.3 Characterizing the dynamic infection microbiome and metatranscriptome

Conventional microbiome and metatranscriptome analyzes—such as short−read sequencing and 16S rRNA gene profiling—typically enable taxonomic identification but do not capture microbial transcriptional activity, limiting the ability to differentiate pathogenic drivers from commensal colonizers during infection (Yadav and Pandey, 2022). In parallel, short−read platforms cannot achieve *de novo* assembly of individual strain genomes within complex communities, nor can they elucidate interspecies gene exchange and interaction networks, posing substantial constraints for investigating the ecological architecture and pathogenic mechanisms of polymicrobial infections (Kazantseva et al., 2024).

ONT-based metatranscriptomics, which involves direct sequencing of total RNA from clinical specimens, offers a powerful means to simultaneously profile both community composition and activity (spanning bacteria, viruses, and fungi) together with *in situ* gene expression patterns (Liu and Wang, 2025; Hempel et al., 2022). This approach is particularly transformative for deciphering polymicrobial infections such as pediatric pneumonia. By revealing which taxa exhibit transcriptional activity during disease, it enables a critical distinction between actively pathogenic organisms and passive commensals. Moreover, ONT metatranscriptomics can uncover dynamic inter−species interactions; for instance, it may detect how viral co−infection induces upregulation of bacterial adhesion factors, thereby intensifying pathogenesis.

Its utility is well illustrated in neonatal studies, where it has been employed to monitor gut microbiome transcriptional responses to clinical interventions such as antibiotics and probiotics, directly linking shifts in microbial community dynamics to the risk of neonatal sepsis (Healy et al., 2025; Keshet et al., 2025).

## 5 Key challenges in ONT clinical translation

The bioinformatics of Oxford Nanopore Sequencing is the central bridge connecting raw sequencing data to clinical diagnostic results. Although promising, significant translational hurdles must be overcome before ONT can be transformed from a powerful research platform to a routine pillar of clinical diagnostics.

### 5.1 ONT Bioinformatics analysis processes and tools

#### 5.1.1 Data generation and quality control: real−time or quasi−real−time base identification

Real−time or quasi−real−time base calling constitutes the initial step of the ONT analytical pipeline. Raw electrical signals generated by the sequencer (stored in FAST5 format) must be converted into nucleotide sequences (FASTQ) before downstream processing (Du et al., 2021). At present, the latest official base−caller, Dorado (v5.0+), is recommended as the primary tool, owing to its markedly improved accuracy and processing speed, as well as native support for GPU acceleration. While Guppy remains in widespread use—particularly for compatibility with legacy workflows and for detection of base modifications such as 5mC and 6mA—the developers advocate migration to Dorado for new projects.

Primary quality control (QC) of raw reads is essential to ensure data reliability. Utilities such as NanoPlot, NanoStat, and PycoQC (components of the NanoPack toolkit) can be employed to evaluate per−run metrics including yield, read length distribution, Q−scores, and signal integrity. These QC assessments provide a critical foundation for subsequent filtering strategies and inform decisions regarding dataset suitability for specific analytical objectives.

#### 5.1.2 Pathogen identification and classification

##### 5.1.2.1 Taxonomic analysis tools

Taxonomic assignment and abundance quantification for metagenomic or 16S amplicon datasets are typically performed using specialized classifiers. Kraken2 and Centrifuge are frequently employed as first−line tools owing to their rapid processing and high classification accuracy (Hiseni et al., 2021; Chen et al., 2026). Kraken2 utilizes a k−mer–based algorithm optimized for ONT long−read data, delivering reliable species identification for common pathogens (e.g., Escherichia coli, Pseudomonas aeruginosa). Centrifuge offers greater memory efficiency than Kraken2, rendering it suitable for large−scale sample sets and enhancing sensitivity for low−abundance taxa.

For full−length 16S/ITS amplicon data, EasyAmplicon2 is specifically designed to accommodate ONT and PacBio reads; it integrates the SILVA and GTDB reference databases and supports an end−to−end analytical workflow with over 30 visualization modules (Luo et al., 2026). Minimap2 serves as a versatile mapper for aligning reads against custom pathogen reference collections, particularly advantageous for targeted analysis of clinically suspected high−priority pathogens (Bosilj et al., 2024). In such applications, the currency and taxonomic specificity of the reference database—such as the regularly updated NCBI nt/nr and RefSeq repositories—are critical determinants of analytical performance.

##### 5.1.2.2 Databases

Diagnostic reliability hinges on the use of high−quality, curated reference databases. Local implementations should incorporate and tailor resources such as NCBI Pathogen Detection, NCBI RefSeq, and specialized viral and fungal collections (e.g., CBS), aligned with regional epidemiological profiles to maximize detection accuracy and relevance (Sayers et al., 2022).

#### 5.1.3 Antimicrobial resistance databases and analysis tools

This module focuses on the identification and functional annotation of antimicrobial resistance genes (ARGs) and their genetic contexts. CARD (Comprehensive Antibiotic Resistance Database) is the most extensive ARG repository, integrating resistance mechanisms, variant alleles, and gene families, and supporting localized BLAST−based searches (Lipworth et al., 2024). ResFinder specializes in horizontally acquired resistance genes (>3,000 sequences) and incorporates PointFinder for mutation−level resistance detection.

Commonly used analytical pipelines include:

ARGs−OAP — an automated workflow that combines CARD and ResFinder, primarily optimized for short−read data with limited compatibility for ONT long reads. DeepARG — a deep−learning−based predictor delivering high specificity for ARG identification in long−read datasets. AMRFinderPlus — developed by NCBI for genome annotation and linkage of predicted resistance loci to phenotypic drug−resistance profiles.

A key advantage of ONT long−read sequencing is its ability to capture the complete physical linkage of ARGs with mobile genetic elements such as plasmids and transposons, thereby overcoming the “gene–host decoupling” limitation inherent to short−read technologies. This capability enables precise reconstruction of resistance gene cassettes and their genomic environment, facilitating mechanistic studies of AMR dissemination (Wang et al., 2025).

#### 5.1.4 Genome assembly and typing

*De novo* assembly of complete genomes in the absence of a reference sequence is a cornerstone of ONT−based pathogenomics. Flye is a widely adopted long−read assembler that handles complex repeats with high robustness, making it well suited for bacterial and fungal genome reconstruction. Shasta, developed by ONT, is optimized for ultra−rapid assembly, capable of completing bacterial genome drafts in under five minutes under optimal conditions. hifiasm−meta, released in 2024, represents a major advance by incorporating ONT Simplex reads to achieve telomere−to−telomere (T2T) assemblies, substantially lowering computational demands while retaining high contiguity—ideal for metagenomic and complex polyploid genome analyzes. wtdbg2 offers a cost−effective alternative that balances assembly speed with contig continuity, rendering it suitable for deployment in resource−limited settings.

Strain typing is essential for delineating phylogenetic relationships and tracing outbreak origins. High−resolution typing tools such as mlst (multilocus sequence typing), chewBBACA (core genome multilocus sequence typing, cgMLST), and Snippy (reference−based SNP calling) enable precise discrimination of strain backgrounds and are integral to molecular epidemiology investigations (Bogaerts et al., 2025) (**Table 2**).

**Table 2:** Summary table of core bioinformatics tools for clinical translation of Oxford Nanopore long-read sequencing.

Analysis phase	Software/ tools	Core features and instructions	Advantages
Base recognition (Base Calling)	Dorado	The official new generation base recognition tool at ONT, which converts fast5 signal to fastq and supports modification detection such as 5mC/6mA	The speed and accuracy are better than Guppy, support GPU acceleration, and v5.0+ version has become the mainstream of the industry
	Guppy	Early official tool that supports flip-flop patterns with RNA modification recognition	Mature and stable, suitable for old processes or specific research scenarios
Taxonomic analysis	Kraken2	Rapid species classification based on k-mer for metagenomic or pathogen detection	It has high recognition accuracy for common microorganisms such as Escherichia coli and Mycobacterium tuberculosis
	Centrifuge	Memory optimized classifier, suitable for large-scale samples with low abundance species detection	It is more memory efficient than Kraken2 and suitable for resource-constrained environments
	EasyAmplicon 2	Full-process 16S/ITS amplicon analysis tool with integration of SILVA/GTDB database and support for ONT/PacBio	Provides end-to-end analysis with 30+ visualization charts, suitable for microbial community research
Antimicrobial resistance (AMR) analysis	CARD	The most comprehensive ARG database	Contains resistance mechanisms, mutation sites, gene families and supports local BLAST alignment
	ResFinder	Resfinder focuses on acquired resistance genes with point mutations (such as gyrA mutations), supporting plasmid origin tracing	The database is updated frequently and is suitable for clinical drug resistance phenotype prediction
	AMRFinderPlus	Developed by NCBI to integrate gene annotation with phenotype association and support automatic reporting	It is used by the FDA for genomic monitoring and is suitable for clinical and regulatory applications
	DeepARG/ARGs-OAP	Deep learning models or automated processes for high-throughput ARGs prediction	DeepARG has a strong generalization ability for unknown drug resistance genes
Genome Assembly (*De novo*)	Flye	Long-read assembler based on repeat graphs, good at handling complex repeat regions	It is widely used in bacterial, fungal, animal and plant genomes because of its high assembly continuity
	Shasta	Officially developed by ONT, designed for real-time surveillance of clinical pathogens	Suitable for real-time pathogen monitoring and field deployment
		
	hifiasm-meta	A tool that combines ONT Simplex data to enable T2T (telomere to telomere) assembly	Complete maps can be obtained without HiFi data, significantly lowering the computational threshold
	wtdbg2	Efficient assembler that balances speed with accuracy	Low memory footprint for large-scale or resource-constrained projects

#### 5.1.5 Data processing workflow: nf−core/nanoseq

The nf−core/nanoseq pipeline represents the current benchmark for automated analysis of ONT transcriptomic and genomic data. Built on Nextflow with Docker containerization, it ensures reproducibility and scalability across computing environments. The workflow comprises four principal stages: Raw data preprocessing-demultiplexing with qcat and removal of contaminant sequences using NanoLyse. Quality control-filtration of low−quality reads (Q < 7, length < 500 ;bp) via NanoFilt, followed by visualization of read length distributions and Q−scores with NanoPlot (Liu et al., 2022). Sequence alignment -mapping to a reference genome (or transcriptome) using Minimap2. Variant detection and transcriptome analysis -for short variants, Medaka is recommended; DeepVariant exhibits limited compatibility with ONT long−read data. Structural variant detection can be performed with Sniffles2. For RNA data, transcript reconstruction and quantification are carried out using Bambu or StringTie2.

#### 5.1.6 ONT clinical workflow

The whole process is divided into three core stages: sample input, wet laboratory processing and dry laboratory analysis. The final standardized clinical report is generated. The specific process includes sample input, wet laboratory processing, dry laboratory analysis and standardized clinical report generation.

**Figure 4:**
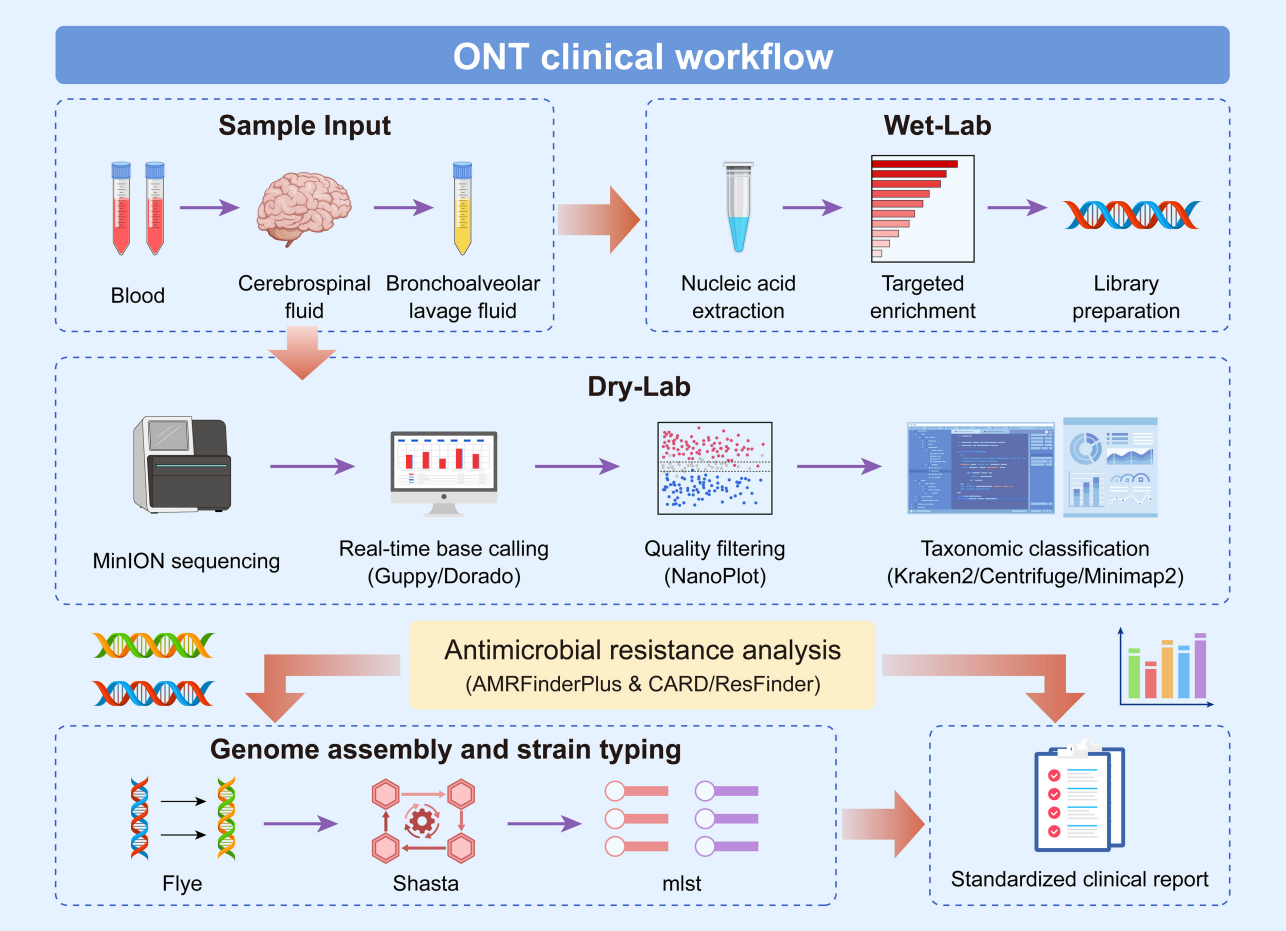
The ONT clinical workflow can be carried out in the order of sample input, wet-lab experiments, dry-lab experiments, special analyzes and report output: first, collect clinical samples such as blood, cerebrospinal fluid and bronchoalveolar lavage fluid, then complete nucleic acid extraction, target enrichment and library preparation in the wet-lab experiments; subsequently, in the dry-lab stage, perform sequencing with the MinION device, use Guppy/Dorado tools for real-time base calling, filter out low-quality data with NanoPlot, and finally conduct classification analysis using tools such as Kraken2, Centrifuge and Minimap2; then carry out special analyzes, on the one hand, complete resistance analysis by combining the AMRFinderPlus tool with the CARD/ResFinder databases, and on the other hand, use Flye and Shasta successively for genome assembly and mlst for strain typing; finally, generate a standardized clinical report to provide a basis for clinical diagnosis.

The whole process is divided into three core stages: sample input, wet laboratory processing, and dry laboratory analysis. Finally, standardized clinical reports are generated. Then entered the stage of dry experiment, first by sequencing MiniON sequencer, reoccupy Guppy real-time identification/Dorado software base sequence, after using NanoPlot software filtering low quality data, reusing Kraken2/Centrifuge Minimap2 analysis tools such as taxonomy, At the same time, AMRFinderPlus and CARD/ResFinder tools were used to detect antimicrobial resistance genes. Finally, Flye, Shasta, mlst and other software were used to complete genome assembly and strain typing. Finally, all analysis results were integrated and standardized clinical reports were generated to provide a basis for clinical diagnosis and treatment (**Figure 4**).

### 5.2 Standardization and optimization of bioinformatics analysis process

ONT sequencing generates vast volumes of raw signal data, which can create computational bottlenecks. In addition, considerable variability in analytical workflows across laboratories limits the comparability and reproducibility of results. Prior studies have demonstrated lossless compression of signal data by discarding the three least−significant bits—representing predominantly stochastic noise—without compromising downstream base calling or modified base detection, thereby reducing file sizes by ~50% and markedly enhancing data accessibility and portability (Jayasooriya et al., 2025).

On the analytical pipeline front, an optimized fast barcoding strategy combined with the Dorado suite (v5.0.0) for base calling, error correction, and assembly has achieved high accuracy without requiring short−read or reference−genome correction. This approach yields genome−wide consensus and core−genome multilocus sequence typing (cgMLST) clustering results comparable to those obtained with Illumina platforms (Vereecke et al., 2025). Furthermore, the CADECT computational pipeline was developed to identify and remove chimeric sequences, substantially improving contiguity in assemblies derived from degraded DNA samples (Agyabeng-Dadzie et al., 2025).

Nevertheless, a unified bioinformatics standard remains lacking, particularly for metagenomic contexts where efficient discrimination between pathogen and host sequences and accurate annotation of antimicrobial resistance genes and virulence factors are essential. There is a pressing need to establish automated, end−to−end analytical frameworks that have been rigorously validated across multiple centers to ensure consistent, reproducible outcomes.

### 5.3 Raw sample processing and optimization of nucleic acid extraction

In pediatric emergency settings, raw clinical specimens-such as blood and respiratory secretions-often contain a low pathogen burden amid a high background of host nucleic acids, posing substantial challenges for both extraction efficiency and nucleic acid purity. Selective enrichment strategies combined with ONT sequencing have been shown to markedly enhance detection of low−abundance pathogens and to improve the quality of metagenomic assembled genomes (MAGs), yielding 86 high−quality MAGs, including 70 pathogen−derived MAGs (Fu et al., 2023).

Moreover, for samples with extremely limited DNA input, multiple displacement amplification (MDA) coupled with ONT rapid library preparation can successfully generate near−complete genome sequences—spanning Gram−positive bacteria, Gram−negative bacteria, and fastidious eukaryotic pathogens—from as little as 0.025 ;ng of total DNA (Agyabeng-Dadzie et al., 2025).

Nevertheless, many of these optimized protocols remain contingent upon specific experimental conditions. Further efforts are required to streamline workflows, reduce pre−processing time, and ensure method versatility and robustness across diverse sample types (e.g., blood, sputum, cerebrospinal fluid) commonly encountered in emergency diagnostics.

### 5.4 Strategy for sequencing accuracy and error rate control

Despite continuous improvements in chemistry reagents and base−calling algorithms, the relatively high sequencing error rate of ONT remains a major barrier to clinical translation. Our analyzes demonstrate that most ONT−only assembled genomes yield robust results in high−resolution typing when using the R10.4.1 flow cell and V14 Rapid Barcoding Kit. However, specific methylation motifs (e.g., GAAGAC) can induce localized misassembles containing >20 erroneous sites, thereby compromising clonal clustering accuracy (Biggel et al., 2024).

A separate long−term outbreak surveillance study similarly reported that base−calling errors led to the inadvertent exclusion of outbreak−associated strains, attributable to interference from proximal methylation modifications (Lohde et al., 2024). To mitigate these challenges, several error−correction strategies have been proposed. One approach couples PCR−based sequencing with masking steps implemented in the MPOA bioinformatics pipeline (Lohde et al., 2024). Another integrates CLAE technology to enhance amplification efficiency and sequencing fidelity of long DNA templates, achieving up to Q30 accuracy for 27% of reads (Yu et al., 2025).

Additionally, the CAPTORs adapter system enables systematic evaluation and correction of sequencing errors, improving the accuracy of detecting pathogenic variants such as BRCA1/2 (Gunter et al., 2022). This strategy holds promise for extension to pathogen detection, potentially enhancing diagnostic precision in infectious disease genomics.

### 5.5 Adaptation of cost−effectiveness analysis to medical insurance policies

Although ONT offer advantages in portability and low hands−on operational cost, its overall cost−effectiveness in pediatric emergency care requires systematic evaluation. A clinical study of 16S rRNA sequencing reported a per−test cost of approximately US$25.30 for ONT (24−sample multiplexing), markedly lower than US$74 for Sanger sequencing, with a correspondingly shorter turnaround time (Campodónico et al., 2025; Zhang et al., 2023).

In practice, NTS can deliver results within ~7 ;h, achieving a pathogen detection rate of 69.5%-comparable to metagenomic mNGS (74.7%) and substantially higher than blood culture (33.9%). Crucially, NTS enables timely, etiology−guided antibiotic adjustment, with an effective response rate of 93.02% for antimicrobial therapy (Zhang et al., 2023; Han et al., 2024).

Nevertheless, current medical insurance schemes rarely reimburse such emerging molecular diagnostic modalities, thereby constraining their routine implementation. Moreover, while the upfront capital investment for ONT equipment is relatively modest, comprehensive cost assessments must account for hidden expenditures, including reagents and consumables, bioinformatics infrastructure and support, and specialized personnel training. Future efforts should focus on conducting real−world cost−effectiveness analyzes and fostering policy development to facilitate appropriate adoption and sustainable integration of ONT−based diagnostics in emergency pediatric infection management.

## 6 Future development direction and outlook

### 6.1 Development of automated sample pre−processing systems

Current implementation of ONT for pathogen diagnostics in pediatric emergency departments remains heavily reliant on manual sample pre−processing steps-including nucleic acid extraction, library construction, and target enrichment-which not only prolongs overall turnaround time but also elevates the risk of procedural error. To realize truly “point−of−care rapid diagnosis,” there is an urgent need for integrated, automated sample pre−processing platforms.

Previous studies have demonstrated that multiple displacement amplification (MDA) combined with ONT rapid library preparation can yield high−quality pathogen genome assemblies from as little as 0.025 ;ng of total DNA input, while the CADECT tool effectively removes chimeric sequences to enhance assembly continuity (Agyabeng-Dadzie et al., 2025). In addition, targeted enrichment strategies have been shown to markedly improve pathogen detection rates and the quality of metagenomic assembled genomes (MAGs) in complex samples; for example, 86 high−quality pathogen MAGs were recovered from drinking water specimens, far surpassing the 12 MAGs obtained by direct sequencing (Fu et al., 2023).

Looking ahead, integration of microfluidic chips, magnetic−bead purification, and PCR−free library construction technologies should be promoted to establish an end−to−end automated workflow that seamlessly converts raw clinical specimens (e.g., blood, sputum, cerebrospinal fluid) into sequencing−ready libraries. Such systems would minimize human intervention, shorten turnaround time, and enhance reproducibility, thereby meeting the demand for high−throughput, high−stability diagnostics in pediatric emergency care.

### 6.2 Artificial intelligence−assisted real−time data analysis

A core advantage of the ONT platform lies in its real−time sequencing capability. However, the sheer volume and complexity of raw signal data impose substantial demands on bioinformatics processing. In recent years, deep−learning algorithms have demonstrated remarkable potential for enhancing base calling, variant detection, and methylation analysis.

For instance, integration of the Dorado SUP v0.9.0 algorithm for baseline correction with Flye assembly enabled respiratory pathogen genome error rates to be reduced to <0.5% at coverage depths ≥35×, with cgMLST typing accuracy comparable to Illumina gold−standard references (Zidane et al., 2025). Another study leveraged deep−learning−based variant calling to improve genotyping accuracy on the R10.4 flow cell, albeit at the cost of increased computational burden due to local haplotype inference (Kolesnikov et al., 2023). Furthermore, a reference−free bioinformatics pipeline, MPOA, has been developed to identify and mask problematic regions arising from systematic sequencing errors linked to GAAGAC methylation motifs (Lohde et al., 2024).

Looking ahead, there is a pressing need to further embed artificial intelligence into ONT workflows, developing lightweight, edge−computing−compatible real−time analysis engines. Such systems would support on−device pathogen identification, antimicrobial resistance prediction, and mixed−infection profiling directly on portable instruments such as MinION, thereby closing the loop for a seamless “sequencing−to−diagnosis” workflow.

### 6.3 Integration pathway of miniaturized devices in the emergency department

Miniaturized sequencing platforms such as Oxford Nanopore Technologies’ MinION have proven valuable in resource−limited settings, enabling whole−genome sequencing of SARS−CoV−2 without reliance on external power or internet connectivity, thanks to their portability, low energy consumption, and standalone operability. A single run can generate 289 near−full−length genomes in 9 ;h ;15 ;min (Park et al., 2024), underscoring the feasibility of deploying such devices at the point of care. These attributes make MinION particularly well suited for integration into pediatric emergency units.

Nevertheless, routine implementation in emergency departments faces several barriers, including lack of standardized workflows, spatial constraints, and insufficient staff training. To facilitate deep integration, it is essential to design purpose−built workstations that consolidate sample processing, sequencing, and analytical modules, and to establish standardized operating procedures (SOPs). Concurrently, interoperability with electronic medical record (EMR) systems should be developed to enable automated result delivery and clinical decision support. Ultimately, these measures will underpin a “sample−in, report−out” rapid response system for emergency care.

### 6.4 Recommendations for designing multicenter clinical validation studies

Although numerous single−center studies have demonstrated the high sensitivity and specificity of ONT for detecting pediatric infectious pathogens—for example, NTS identified causative agents in 90% of blood samples from patients with severe infections (Zhang et al., 2023)—its clinical translation still lacks large−scale, prospective, multicenter validation.

Future investigations should adopt harmonized sample types (e.g., whole blood, cerebrospinal fluid, sputum), standardized library−preparation protocols (e.g., V14 Rapid Barcoding Kit (Biggel et al., 2024)), and consensus bioinformatics pipelines (e.g., ONT−only workflows based on Dorado kits (Vereecke et al., 2025)). Inclusion of study sites across diverse geographic regions and healthcare resource settings is essential, with evaluation focused on timeliness (target < ;8 ;h) (Goenka et al., 2022), cost−effectiveness, and impact on clinical outcomes—such as antimicrobial treatment efficacy and mortality—in real−world emergency department settings (Goenka et al., 2022).

In addition, conventional methods (blood culture, PCR panels) and metagenomic mNGS should serve as comparator arms, while concurrent collection of antimicrobial susceptibility data is imperative to assess concordance between genotype−predicted and phenotype−confirmed resistance (e.g., NTS resistance prediction agreed with culture confirmation in 80.6% of cases (Han et al., 2024)). Such rigorously designed studies will provide pivotal evidence to inform the recommendation level of ONT (e.g., whether it meets Class ;II evidence criteria) for infection diagnosis and management in pediatric emergency care.

## 7 Conclusion

ONT sequencing is poised to fundamentally transform pediatric emergency infectious disease management by bridging the critical gap between diagnostic speed and etiological breadth. Its unique capabilities-ultra-long reads for comprehensive pathogen and AMR profiling, real-time analysis enabling results within hours, and unparalleled portability for point-of-care deployment-address the core limitations of conventional methods. Beyond revolutionizing rapid diagnostics for conditions like sepsis and meningitis, ONT opens new avenues for fundamental research into host-pathogen interactions and pathogen evolution. While challenges in bioinformatics and workflow integration persist, its trajectory from a research tool to an essential clinical component is clear, as evidenced by pilot studies demonstrating its feasibility in critical care. Ultimately, integrating ONT into a holistic “precision infectious diseases” framework, which synergizes genomic, phenotypic, and host immune data, holds the promise not only of saving lives through timely, targeted therapy but also of advancing our fundamental understanding of infection biology to improve global child health outcomes.
